# Antibiotic use in poultry farming: a cross-sectional study of veterinary practices in Tunisia

**DOI:** 10.3389/frabi.2025.1646766

**Published:** 2025-10-14

**Authors:** Mehdi Ben Ali, Badi Chtioui, Hamza Bouchrit, Hatem Laamiri, Hedia Attia El Hili

**Affiliations:** ^1^ Independent Researcher, Manouba, Tunisia; ^2^ Commissariat Régional de Développement Agricole Nabeul, Nabeul, Tunisia; ^3^ Independent Researcher, Tunis, Tunisia

**Keywords:** veterinary prescribing, avian medicine, antibiotic resistance, rapid AST, one health, critically important antibiotics, self-medication

## Abstract

Antimicrobial resistance (AMR) in poultry production poses a growing public health threat due to the emergence of multidrug-resistant (MDR) bacteria and the risk of transmission to humans through direct or indirect contact with these germs. In Tunisia, limited data on antibiotic use and veterinary prescribing practices hinder the development of effective AMR mitigation strategies, particularly in a sector with high antibiotic consumption. A cross-sectional study was conducted among veterinarian prescribers in avian medicine in Tunisia to assess their antibiotic prescribing behaviours and related practices and to evaluate their potential contribution to AMR emergence and spread. The most frequently reported first and second-line antibiotics were enrofloxacin (20/52 and 14/52), florfenicol (14/52 and 14/52), and doxycycline (7/52 and 6/52). Colistin (10/52) was the most used third-line antibiotic. These antibiotics were often administered without microbiological confirmation. Although 69% had access to accredited labs, 42% relied on rapid antimicrobial susceptibility tests (RASTs). Waste management practices were inadequate, with 50% disposing of biological waste in regular trash and 42% discarding expired antibiotics into the environment. Additionally, 77% reported frequent farmer self-medication. These findings highlight the urgent need for targeted training, improved surveillance, and the application of the One Health approach to tackle AMR in Tunisia’s poultry sector.

## 1 Introduction

Over the past few decades, the poultry industry has undergone a remarkable expansion worldwide. Poultry meat production has risen from 9 million tonnes in 1961 to more than 144 million tonnes in 2023, illustrating a significant intensification of farming systems (Ritchie et al., 2019; FAO, 2024). However, this expansion has been accompanied by an increase in the health risks associated with viral and bacterial diseases, leading to greater use of veterinary antibiotics for the prevention and treatment of infections (Castanon, 2007; Caneschi et al., 2023; WOAH, 2024).

Global antibiotic use in livestock, including poultry farming, is projected to rise significantly. Estimates indicate that between 2010 and 2030, worldwide antimicrobial consumption will increase by 67%, growing from 63,151 ± 1,560 tons to 105,596 ± 3,605 tons (Van Boeckel et al., 2015). This surge is primarily driven by the growing demand for animal protein worldwide (Abou-Jaoudeh et al., 2024). In poultry production specifically, antibiotics account for a significant portion of this usage, particularly in regions where intensive farming practices are prevalent. For example, in some countries, up to 70-80% of total antibiotic consumption is attributed to livestock, with poultry being one of the largest contributors (Tiseo et al., 2020). This widespread use includes therapeutic, prophylactic, and growth-promoting purposes (WOAH, 2024).

The massive and sometimes inappropriate use of antibiotics in poultry farming has encouraged the emergence and dissemination of resistant bacteria, contributing to the global antimicrobial resistance (AMR) crisis (Nhung et al., 2017; Agyare et al., 2018; Mak et al., 2022; Singh et al., 2025). Avian zoonotic resistant bacteria can spread to humans through direct contact, ingestion of contaminated food, or environmental exposure (Friese, 2024; Kobuszewska and Wysok, 2024; Magalhães et al., 2024). Even non-zoonotic avian bacteria may contribute to resistance by horizontally transferring their resistance genes to human pathogens, exacerbating the public health risk (Johnsborg et al., 2007; Segawa et al., 2024; Napit et al., 2025). According to predictive statistical models, this phenomenon poses a significant public health challenge. In 2019, an estimated 4.95 million (3.62–6.57) deaths were linked to bacterial antimicrobial resistance (AMR), with 1.27 million (95% UI 0.911–1.71) deaths directly attributable to bacterial AMR (Murray et al., 2022). Described as a “silent pandemic”, antibiotic resistance is spreading rapidly through trade, human mobility, wildlife migration and environmental flows, posing a major challenge in terms of health and environmental safety (Allerberger, 2016; Van Boeckel et al., 2019).

In Tunisia, the poultry industry accounts for 34% of animal production and 59% of the animal meats consumed. According to the 2015-2016 poultry census, 6,209 establishments were counted, with an annual production capacity of 43 472 259 chickens (Veterinary Services Report). This sector is the main consumer of veterinary antibiotics, followed by aquaculture. However, the lack of precise data on the quantity of antibiotics used makes it difficult to assess their impact, particularly given the use of certain specialties that are either authorized for other animal species or employed under extralabel conditions.

The uncontrolled use of antibiotics in poultry farming raises concerns about drug residues in foodstuffs, the transmission of resistance genes and resistant bacteria, and environmental contamination via manure and livestock effluents. These contaminants can affect flora and fauna, as well as posing a risk to human health (Chee-Sanford et al., 2009; Marshall and Levy, 2011; Shakoor et al., 2020; Friese, 2024; Napit et al., 2025). In this context, the role of prescribing veterinarians is central to guaranteeing the safety of food of animal origin, and in contributing to “One Health” strategies to tackle antibiotic resistance, notably through the promotion of good antibiotic use practices, biological waste management and raising awareness among livestock farmers (FAO and WHO, 2019).

This study is the first comprehensive national investigation to assess antibiotic use and AMR awareness among poultry veterinarians. Its originality lies not only in its national scope—covering diverse regions and poultry production systems across Tunisia—but also in its in-depth exploration of veterinarians’ awareness and sensitivity to the phenomenon of AMR. The study goes beyond simply evaluating antibiotic prescribing behavior, aiming also to assess the potential for behavioral change regarding antibiotic use, as well as other management practices that may contribute to the emergence and spread of resistance within environment. By evaluating the alignment between field-level practices and AMR awareness, this research offers valuable insights into the veterinary dimension of the One Health approach, which remains underexplored in the region. The findings aim to support evidence-based interventions for antimicrobial stewardship, tailored to the specific needs and challenges of the Tunisian poultry sector.

Our investigation aims to describe and assess the antibiotic prescribing and management practices of veterinarians working in poultry production, and to evaluate how these practices may influence the emergence and spread of AMR.

## 2 Results

### 2.1 Socio-demographic characteristics of respondents

**Figure 1:**
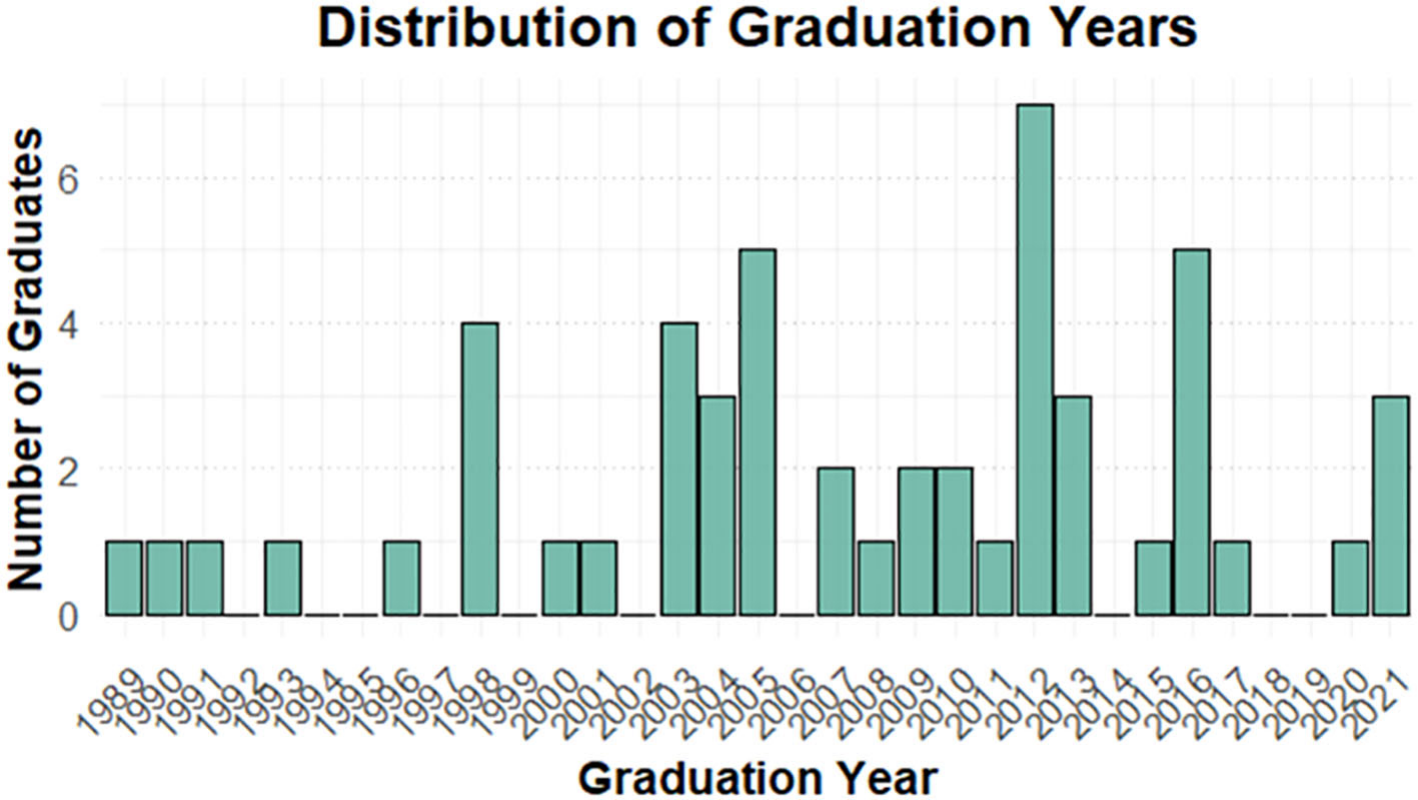
Distribution of respondents’ graduation years.

In this study, 52 of the 76 avian veterinarians practising nationwide took part in the survey, yielding a response rate of (68%). Most respondents were male (71%) and practised in private clinics (65%). Veterinarians graduating between 2004 and 2013 accounted for the largest proportion (50%), with graduation years ranging from 1989 (oldest) to 2021 (most recent) (**Figure 1**).

In terms of professional experience, the majority of participants (44%) had between 6 and 15 years’ experience, closely by those with more than 15 years’ experience (40%), resulting in a mean experience of 14 years. With regard to training on AMR, (58%) of respondents said they had received training, of which (27%) (n=30) had received less than 3 days’ training (**Table 1**).

**Table 1:** Socio-demographic characteristics of respondents.

Variable	Response	Frequency (n = 52)	Percentage (%)
Gender	Female	15	29
	Male	37	71
Work Sector	Private clinic	34	65
	Poultry farming company_Holding	12	23
	OTD (*Office de Terres Domaniale*: State livestock farms)	04	08
	Other	02	04
Years since graduation	1989-2003	15	29
	2004-2009	13	25
	2010-2013	13	25
	2014-2021	11	21
Years of experience	1-5	08	16
	6-15	23	44
	>15	21	40
Training on AMR	Yes	30	58
	No	22	42

### 2.2 Frequently prescribed antibiotics in avian veterinary practice

**Figure 2:**
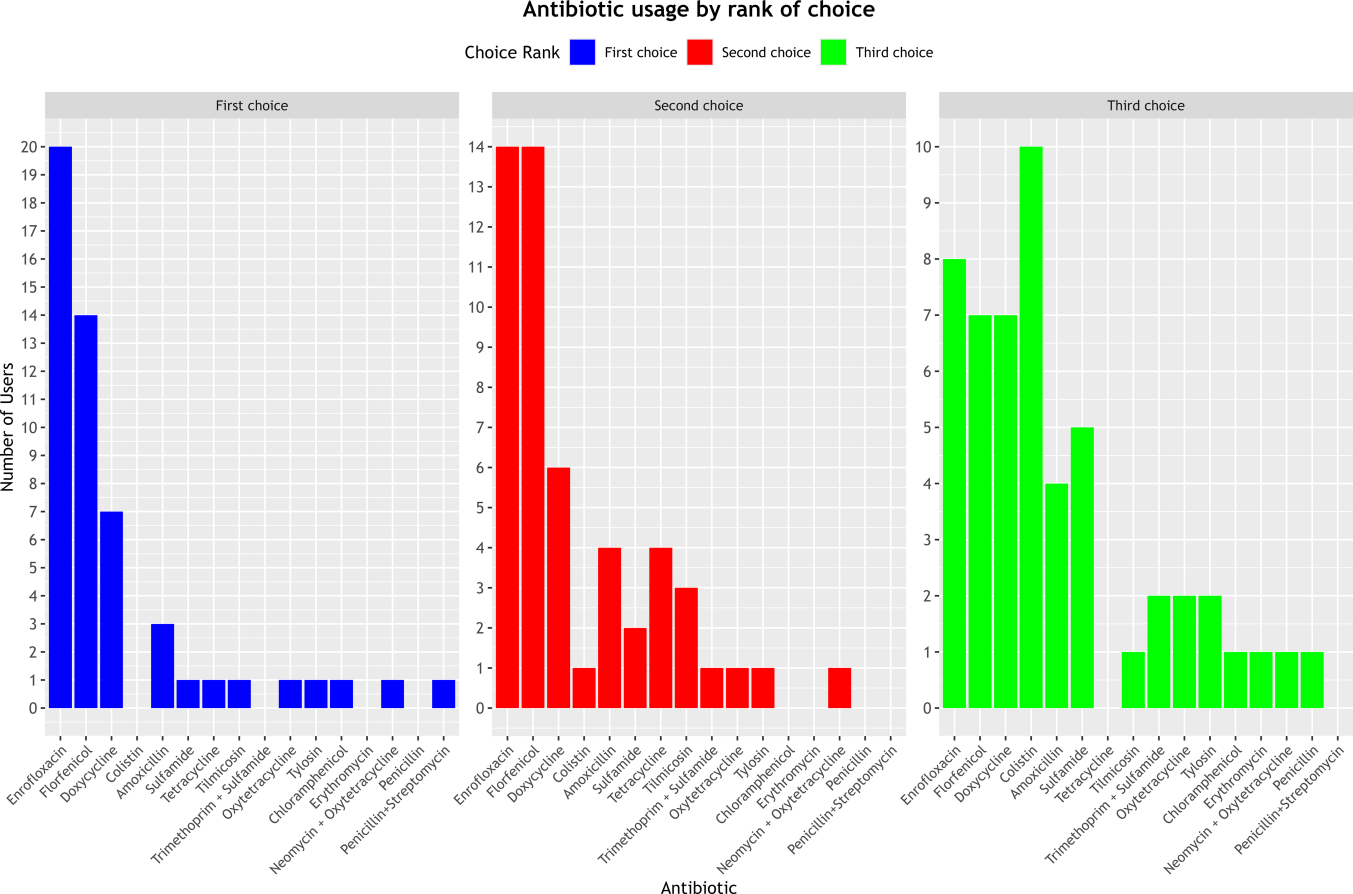
Ranking of prescribed antibiotics based on order of choice.

The most commonly cited antibiotics as first and second-choice treatments were enrofloxacin, florfenicol, and doxycycline, reported in (20/52), (14/52), and (7/52) cases as the primary choice, and (14/52), (14/52), and (6/52) cases as the secondary choice, respectively. For third-line therapy, the most frequently used antibiotics were colistin (10/52), enrofloxacin (8/52), and florfenicol and doxycycline (7/52 each) (**Figure 2**). However, respondents did not provide data on the approximate annual quantities of these prescribed antibiotics.

**Figure 3:**
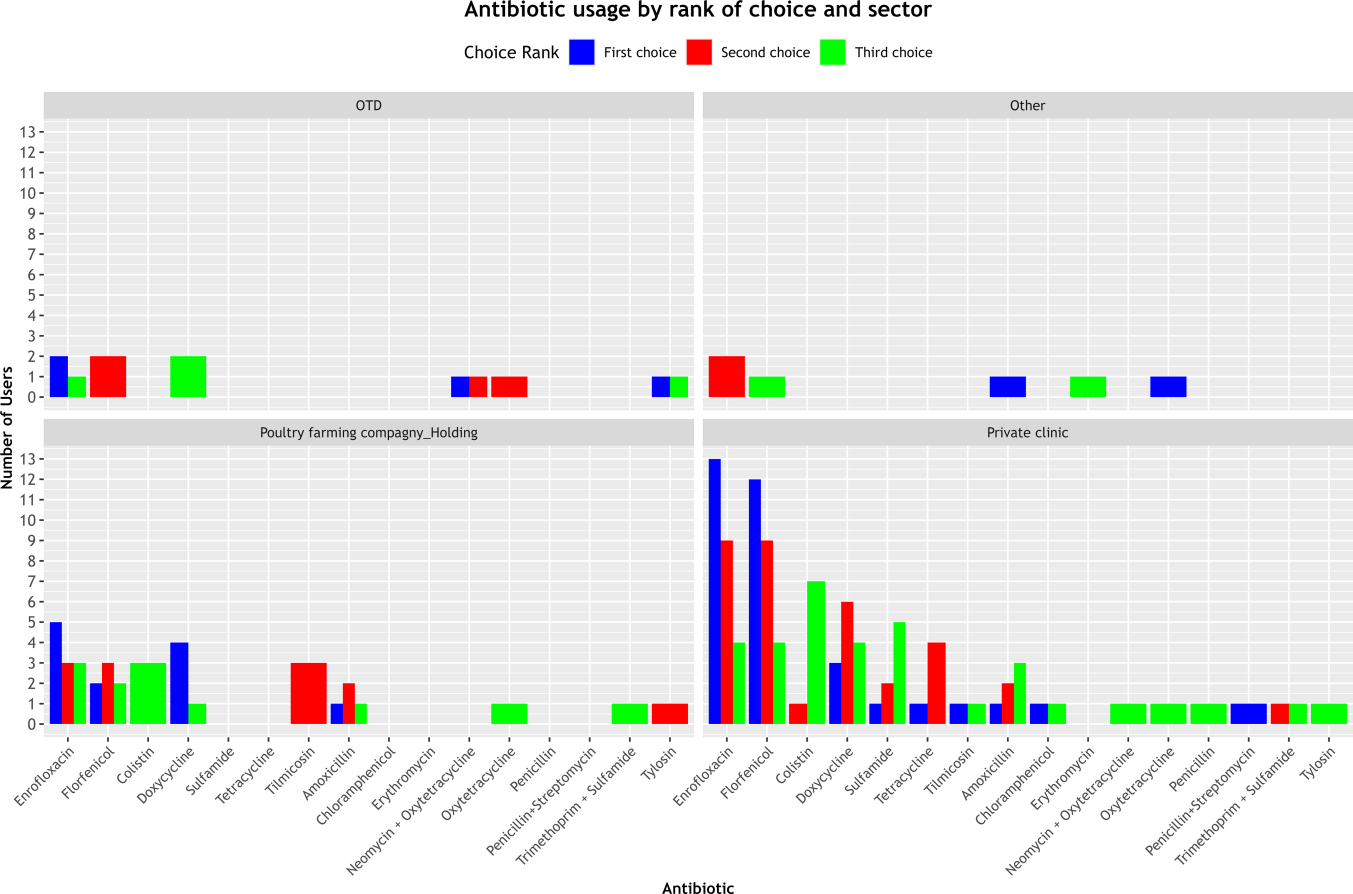
Classification of prescribed antibiotics by choice order and work sector.

**Figure 3** shows a marked variation in the antibiotics selected across the three choice ranks between different sectors of activity.

### 2.3 Antibiotic use practices

According to survey respondents, the most frequently used route of administration for avian medicine is per os (96%). The most common prescription durations were 5 days, [4 to 5days] and [3 to 5days], with (27%), (21%) and (15%) respectively. With regard to the symptoms justifying the systematic use of antibiotics, the most frequently mentioned answers were the combination of respiratory and digestive symptoms (56%) and isolated respiratory symptoms (25%). The choice of first-line antibiotic was based mainly on the result of the rapid antibiotic susceptibility testing (RAST) using the disk diffusion method directly from clinical swabs on Müller-Hinton agar and results read within 24 hours (17%); on a combination of RAST, the farm’s history, and the veterinarian’s experience (12%); and on the veterinarian’s experience alone (10%). The majority of veterinarians surveyed (68%) use antibiotic prophylaxis, with (46%) of them doing so frequently. Statistical analyses in **Table 2** revealed significant associations between prophylactic antibiotic use and several factors, including years of experience (p=0.03), work sector (p=0.04), and graduation year (p=0.03).

**Table 2:** Statistical association between antibiotic misuse practices and sociodemographic factors.

Variables	Training on AMR			Off-label use			Antibiotic prophylaxis			Laboratory use			Automedication		
	Yes	No	P value	Yes	No	P value	Yes	No	P value	Yes	No	P value	Yes	No	P value
Sex			1			0.2099			0.0894			0.825			
Female	09	06		01	14		08	07		14	01				
Male	21	16		10	27		30	07		32	05				
Years of experience			0.556			0.4641			0.0324			0.4304			
1-5	04	04		02	06		03	05		06	02				
6-15	12	11		05	18		17	06		21	02				
>15	14	07		02	19		18	03		19	02				
Work sector			0.2193			0.2607			0.0453			0.4987			2.098e-06
Private clinic	18	16		09	25		28	06		28	06		33	01	
Poultry farming company_Holding	09	03		01	11		07	05		12	0		04	08	
OTD	01	03		0	04		03	01		04	0		01	03	
other	02	0		01	01		00	02		02	0		02	0	
Years since graduation			0.3969			0.7155			0.03516			0.3855			
1989-2003	11	04		02	13		11	04		15	0				
2004-2009	06	07		03	10		13	0		11	02				
2010-2013	08	05		04	09		08	05		11	02				
2014-2021	05	06		02	09		06	05		09	02				
Training on AMR						0.5609			0.7889			0.0848			
Yes				05	25		21	9		29	1				
No				06	16		17	5		17	5				

Finally, (21%) of respondents indicated that they use antibiotics outside the indications of the marketing authorization (MA). The most frequently cited reasons were the absence of a therapeutic alternative combined with efficacy (5/11), and the absence of a therapeutic alternative alone (3/11).

### 2.4 Microbiological diagnostic practices and laboratory waste management

#### 2.4.1 Use of laboratory tests

The use of accredited microbiology laboratories was reported by (69%) of the surveyed veterinarians, with (52%) using them frequently and (17%) occasionally. The main reasons cited for not using or rarely using these laboratories included distance (5/14) and result turnaround time in relation to the urgency of cases (3/14).

A significant proportion of respondents (42%) had their own laboratory facilities for RAST.

Regarding the management of biological waste generated during their activities, the majority stated that they pre-treated the waste before disposal, either in designated biological waste bins (50%) or in regular bins (29%).

The management of necropsy-related waste (e.g., carcasses, gloves) varied among respondents: (38%) reported leaving it with the farmer or discarding it in household waste, while (27%) reported burying it on-site.

#### 2.4.2 Pharmaceutical waste management

As for the disposal of empty or expired antibiotic vials, (46%) of surveyed veterinarians reported placing them in special waste bins, while (42%) admitted to discarding them directly into the environment.

#### 2.4.3 Evaluation of antimicrobial resistance in poultry

In relation to antimicrobial resistance, a large majority (79%) of respondents reported having encountered multidrug-resistant bacteria (MDRB), with (31%) frequently and (48%) occasionally. Among those frequently confronted with MDRB, four reported rarely using accredited microbiology laboratories, and one had never used them. Additionally, seven had never received training on AMR.

Within the (21%) who had never encountered MDRB, five had rarely consulted a laboratory, four had never done so, and eight had not received any training on AMR.

In response to the question regarding the three most frequently encountered MDRB and the antibiotics to which they were resistant, the data were often missing or incomplete. Nevertheless, *Escherichia coli* was the most frequently reported organism.

### 2.5 Antibiotic self-medication in poultry

The unsupervised use of antibiotics by poultry farmers or farm managers without a veter-inary prescription was noted by (77%) of practitioners, with (67%) characterizing it as a frequent practice. A statistically significant association was observed with work sector (p = 2.098 × 10^-6^) (**Table 2**).

The main reasons cited for this practice were the easy access to antibiotics alone (43%) or in combination with a lack of awareness of the associated risks (32%).

According to the respondents, this easy access was primarily linked to the sale of antibiotics without prescription (60%), and in some cases, to antibiotic smuggling (37%).

## 3 Discussion

The 68% participation rate observed in our study is similar to the response rate obtained in a survey of poultry veterinarians in Nepal, although the latter was a KAP (Knowledge, Attitudes, and Practices) study specifically assessing antimicrobial use and antimicrobial resistance in poultry (Shahi and Jeamsripong, 2024). The high participation rate for an online survey, combined with its targeting of the entire population of poultry veterinarians, suggests reasonable representativeness of the study population. Nevertheless, the risk of non-response bias remains, particularly in the absence of data to analyze the profile of non-respondents, which is inherent to the anonymous nature of the survey. It is therefore important to consider that certain characteristics of non-respondents may differ from those of participants, which could influence the study’s findings (Roush, 1998; Groves and Couper, 2012). In addition, potential recall bias and the inherent limitations of self-reported data may have affected the accuracy of responses, particularly with regard to the frequency and context of antimicrobial use. Similarly, questions related to the management of biological and pharmaceutical waste may be subject to reporting bias, as respondents could underreport or misrepresent actual practices. These limitations should be taken into account when interpreting the study’s results.

In our study, enrofloxacin, florfenicol, and doxycycline were the most frequently prescribed antibiotics as first- and second-line treatments by the surveyed practitioners. Notably, colistin was identified as the predominant third-line option, surpassing enrofloxacin in this category. These findings are consistent with patterns observed in other countries. For instance, a large-scale cross-sectional study conducted in 2021 in Bangladesh by Chowdhury et al. reported that the most commonly used antibiotics in commercial chicken production were oxytetracycline (23–31%, depending on production type), doxycycline (18–25%), ciprofloxacin (16–26%), and amoxicillin (16–44%) (FAO, 2024). Similarly, in a 2011 study carried out in Ogun State, Nigeria, Oluwasile et al. found that poultry farms frequently used Neoceryl R—a commercially formulated broad-spectrum antibiotic containing neomycin, erythromycin, oxytetracycline, streptomycin, and colistin—as well as enrofloxacin and furazolidone (Oluwasile et al., 2014). In Algeria, a survey conducted between 2019 and 2020 in the Ain Defla province revealed that quinolones were the most widely used class of antimicrobials (24.4%), followed by tetracyclines (22.5%), sulfonamides (20.1%), and polypeptides (12.1%) (Chowdhury et al., 2022). These comparative findings highlight a global trend of extensive antimicrobial use in poultry production, often involving critically important drugs, thereby raising serious concerns about the potential acceleration of antimicrobial resistance. Enrofloxacin is widely used as a first- and second-line treatment in avian medicine due to its broad spectrum of activity, good oral bioavailability, and particularly its efficacy against the most common and detrimental digestive and respiratory infections in poultry (Oluwasile et al., 2014; Mokhtar Rahmani et al., 2021). Similarly, florfenicol is frequently prescribed for its good oral bioavailability, broad spectrum (including activity against intracellular bacteria), and its recommended use in respiratory infections (Dimitrova et al., 2006). Doxycycline is also extensively employed due to its broad-spectrum coverage and efficacy against intracellular bacteria; it is more frequently prescribed in laying hens due to the absence of a withdrawal period for eggs (Dimitrova et al., 2006). Finally, colistin is often reserved as a third-line treatment, serving as a last-resort option against Gram-negative bacteria—particularly *E. coli* strains resistant to the aforementioned antibiotics. Its use is limited due to its demanding intramuscular administration and the emergence of colistin-resistant Gram-negative bacteria in Tunisia (Mekala et al; Landoni and Albarellos, 2015; Grami et al., 2016; Saidani et al., 2019).

Enrofloxacin and colistin are classified as critically important antibiotics in both veterinary and human medicine. Their use in veterinary practice is strictly regulated by WOAH (World Organisation for Animal Health) guidelines, which prohibit their first-line use, prophylactic administration, or off-label applications. Justification for their prescription requires supporting antibiogram data (Hassen et al., 2020). These restrictions aim to preserve the efficacy of these drugs, which are considered last-resort antibiotics in human medicine (Hassen et al., 2020). Florfenicol and doxycycline, meanwhile, are categorized as critically important antimicrobials in veterinary medicine only (WOAH, 2024). Multiple studies in Tunisia have highlighted a high prevalence of resistance to tetracyclines (WHO; Di Francesco et al., 2021; Di Francesco et al., 2023).

The routine uses of antibiotics—particularly those classified as critically important—without prior bacteriological testing constitutes irrational and potentially harmful antimicrobial use. This practice is especially concerning in the context of poultry production, where parasitic and viral infections are the predominant causes of disease, and antibiotics are often administered primarily to prevent secondary bacterial infections (Lelkes et al., 2012; Badi et al., 2018; Gharbi et al., 2018; Bagra et al., 2023; WOAH, 2024).

The survey also revealed a lack of quantitative data on annual antibiotic consumption, reflecting either sensitivity around the issue or insufficient traceability of these drugs. This gap hinders the assessment of selective pressure exerted by these antibiotics, thereby limiting the implementation and evaluation of action plans to reduce their use. Given the alarming situation in a sector where the risk of antimicrobial resistance dissemination poses a global health threat (FAO and WHO, 2019), there is an urgent need for enhanced and integrated surveillance of antibiotic prescriptions.

The utilization rate of accredited microbiological analysis laboratories is relatively high (69%); however, frequency analysis reveals that nearly half of practitioners rarely or never use them. This compromises the quality of clinical diagnosis and the rational use of antibiotics. Training on antimicrobial resistance appears to be statistically associated with the use of laboratory analysis. Nevertheless, the examination of data on isolated MDR bacteria raises uncertainties regarding this association. Indeed, inconsistencies or the absence of such data may result either from a lack of laboratory utilization or from a poor understanding of the very definition of a MDR bacteria. However, the survey methodology, particularly its anonymous nature and online distribution, does not allow for clarification of these ambiguities (Badi et al., 2018).

Over the past decade, the poultry sector has seen the implementation of bacteriology laboratories within veterinary clinics and agricultural holdings. According to our survey, 42% of respondents have access to such laboratories and perform RAST themselves. This technique, designed for fast-growing bacteria (less than 24 hours), allows them to obtain antibiotic susceptibility profiles within hours without requiring bacterial identification. Although this method aims to promote appropriate antibiotic use, its application may also contribute to misuse by targeting contaminant bacteria or zoonotic pathogens, thereby inducing resistance. To mitigate these potential risks, it is imperative to simultaneously utilize accredited laboratories while ensuring continuous training and awareness-raising among users of this technique.

Among surveyed facilities equipped with laboratories for rapid antibiograms, half dispose of biological waste in regular trash bins. While the majority claim to perform preliminary treatment, others do not apply any neutralization process. Additionally, 38% of practitioners demonstrate inadequate practices regarding autopsy waste management. Concerning pharmaceutical waste, a significant proportion of respondents (42%) take no specific measures and discard these wastes directly into the environment.

These inappropriate practices in the management of biological and pharmaceutical waste reflect a lack of awareness of the environmental dimension of the One Health approach to combating antimicrobial resistance (AMR). Improper disposal of biological and pharmaceutical wastes can release resistant bacteria, resistance genes, and active antibiotic residues into soil and water, posing risks to human, animal, and plant health. Moreover, bacteria released into the environment can interact with plastic debris and other mineral surfaces, facilitating biofilm formation, horizontal gene transfer, bacterial transformation, and increased selective pressure. The gaps identified in current practices—such as inadequate management of pharmaceutical and biological waste and its role in the dissemination of AMR, the frequent use of critically important antibiotics without prior antibiogram testing, and the poor understanding of multidrug-resistant bacteria (as reflected in incorrect responses to questions on bacterial resistance profiles)—will be directly addressed in the design of targeted training modules, ensuring that these critical knowledge and practice deficits are systematically addressed (Lelkes et al., 2012; Gharbi et al., 2018; Bagra et al., 2023; Stevenson et al., 2024; Hendiani et al., 2025).

This situation is particularly concerning given that Tunisia ranks among the countries with the highest environmental pollution scores according to the 2020 Vivid Economics report (Zhu et al., 2019). Improving biological and pharmaceutical waste management practices is therefore crucial to limiting the spread of resistance and preserving the environment. Achieving this goal requires a stringent regulatory framework grounded in recent scientific advances and effectively enforced at the field level. At the policy level, our findings highlight the importance of strengthening legislation, particularly regarding the sale of antibiotics exclusively by prescription, and of implementing effective systems for the collection and safe disposal of biological and pharmaceutical waste. Such measures are essential to reduce antimicrobial misuse and limit the environmental dissemination of resistance. Furthermore, as part of broader practice improvement efforts, it is vital to reinforce AMR surveillance systems—especially through environmental monitoring—and to enhance training programs. Particular attention should be given to priority areas such as the management of biological and pharmaceutical waste and the integration of the One Health approach into AMR mitigation strategies (Pruden et al., 2013; Iskandar et al., 2020; World Economic Forum, 2021).

The final section of this investigation focused on the unregulated use of antibiotics by poultry farmers or farm managers. This practice was reported as highly prevalent (77%) by practitioners, particularly in the informal sector, and was attributed to the over-the-counter sale of antibiotics. A 2024 survey by Shahi and Jeamsripong in Nepal found that 99.1% of participating veterinarians believed that the misuse, inappropriate use, and Non-prescription administration of antibiotics were the primary drivers of antimicrobial resistance (AMR) (Shahi and Jeamsripong, 2024). Similarly, in Pakistan, a study by Habiba et al. (2023) reported a 60% rate of Non-prescription antibiotic use (n=40) (Endale et al., 2023). In Brazil, research by Torres et al. (2022) observed that 11 out of 16 veterinarians working in the egg-laying sector encountered unregulated antibiotic use (Habiba et al., 2023). In Africa, multiple studies conducted in Zambia, Ghana, Tanzania, and Kenya revealed Non-prescription antibiotic sales rates as high as 100% (Nkansa et al., 2020; Azabo et al., 2022; Torres et al., 2022; Kariuki et al., 2023; Mudenda et al., 2024).

This phenomenon will be further explored in an upcoming study targeting poultry farmers’ knowledge, attitudes, and practices (KAP) regarding antibiotic use and the challenges of antimicrobial resistance. Such surveys are critical, given that farmers play a key role in administering antibiotic treatments—whether prescribed or not. Therefore, assessing their understanding of proper antibiotic therapy practices, particularly concerning dosage adherence, treatment duration, and withdrawal periods before marketing, is essential. This study will also specifically examine the underlying causes of self-medication practices, with its findings expected to identify the key drivers of this phenomenon and directly inform the development of tailored awareness-raising and educational activities for farmers.

## 4 Materials and methods

A cross-sectional questionnaire survey was carried out from August 2024 to January 2025 among all veterinarians prescribing in avian medicine in Tunisia. The questionnaires covered a range of topics, including: demographic information, commonly used antibiotics and factors influencing treatment regimens, laboratory practices, antibiotic resistance, biological and pharmaceutical waste management, and self-medication practices.

### 4.1 Survey questionnaire

To gather the required information, a questionnaire was designed using Kobotoolbox. It consists of 44 questions, some of which are displayed only if specific conditions are met in the preceding question. The majority of the questions are semi-open-ended, allowing for a controlled range of responses while still giving respondents the option to provide a non-predefined answer. The approximate time to complete the survey was estimated to be between 10 and 20 minutes.

The questionnaire is structured into four sections: demographic information, commonly used antibiotics and factors influencing treatment regimens, laboratory practices including antibiotic resistance test, biological waste management, and self-medication practices.

The questionnaire is anonymous and distributed via email to all veterinarians prescribing for avian species. It is accompanied by a letter outlining the survey’s objectives and providing a link to the questionnaire. Responses are automatically collected on the Kobotoolbox platform, but only after the respondent has validated and submitted the questionnaire.

To encourage participation, we leveraged Facebook pages dedicated to poultry veterinarians, the *Société Scientifique Tunisienne de Médecine Vétérinaire Aviaire* (SSTMVA), as well as official email channels targeting veterinarians in both public and private sectors.

### 4.2 Questionnaire testing

Prior to launching the survey, an initial version of the questionnaire was tested by five veterinarians with previous experience in poultry practice from both the public and private sectors. These individuals were selected for their familiarity with the field while not being directly involved in current veterinary activities, ensuring objective feedback. Their responses and suggestions were analyzed to refine the questionnaire, leading to the validation of the final version.

### 4.3 Data curation and analysis

The collected questionnaires are imported as an Excel spreadsheet. Once the database is cleaned, it is then analysed using the R software (version 4.2.2). Statistical tests, including Pearson’s Chi-squared test and Fisher’s Exact test, are performed using R and additionally validated using online calculators available on the websites miniwebtool.com (Chi-squared test) and astatsa.com (Fisher’s Exact test). A significance level of 5% (α = 0.05) was adopted for all statistical tests.

## 5 Conclusion

The poultry sector, worldwide and particularly in Tunisia, is the leading consumer of antibiotics in animal production. The collected data highlighted current prescribing practices in avian medicine, including the frequent use of critically important antibiotics in veterinary medicine and sometimes in human medicine too, as first-line treatment or chemoprophylaxis, often without laboratory confirmation.

The study also revealed gaps in the respondents’ knowledge of antibiotic resistance management, especially concerning the environmental dissemination of resistant bacteria and resistance genes. Addressing these deficiencies requires the rapid implementation of continuous training programs tailored to the sector’s needs and based on the One Health approach to combat AMR effectively.

Nevertheless, certain limitations in the survey methodology hindered the collection of key data, including the annual quantities of antibiotics prescribed per veterinarian, the multidrug resistance profiles of the most prevalent bacterial strains in the field, and their resistance spectra.

Finally, the study documented the widespread practice of antibiotic self-medication in poultry, primarily driven by over-the-counter access to antimicrobials without veterinary prescription.

This type of survey should be extended to other livestock sectors, given its ease of implementation and the valuable data it yields. Additionally, targeted studies on farmers’ knowledge and practices related to antibiotic use and antimicrobial resistance are necessary to enhance understanding of the field situation and to educate farmers on best practices in antibiotic therapy, as well as on their critical role in combating antimicrobial resistance.

## Data Availability

The original contributions presented in the study are included in the article/**Supplementary Material**. Further inquiries can be directed to the corresponding author.
