# Supplementary material for: Antibiotic use in poultry farming: a cross-sectional study of veterinary practices in Tunisia
Source: Front Antibiot. 2025 Oct 14;4:1646766. doi: 10.3389/frabi.2025.1646766 (PMC12558877; doi:10.3389/frabi.2025.1646766)
Supplement: Supplementary file 1 [file DataSheet1.pdf]

Utilisation des Antibiotiques en médecine vétérinaire aviaire

Renseignements généraux

Genre

- ◯ Homme
- ◯ Femme

Lieu de travail

- ◯ Cabinet privé
- ◯ Société d'élevage (ou Holding)
- ◯ OTD
- ◯ autre

Précisez

____

Année du diplôme

____

Nombre d'année d'expérience en Médecine aviaire

____

Formation sur l'antibiorésistance

- ◯ Oui
- ◯ Non

Nombre de jours de formation

____

Année de la dernière

____

Organisme de formation

____

Liste des 3 antibiotiques les plus utilisés

Antibiotique 1: Nom & Quantité approximative d'antibiotiques prescrite par an
précisez l'unité

____

Antibiotique 2 : Nom & Quantité approximative d'antibiotiques prescrite par an
précisez l'unité

Antibiotique3 : Nom & Quantité approximative d'antibiotiques prescrite par an
précisez l'unité

Facteurs conditionnant le schéma thérapeutique

Symptômes pour lesquels le recours aux antibiotiques est systématique
sélectionner un ou plusieurs

- [ ] Respiratoires
- [ ] Digestifs
- [ ] Nerveux
- [ ] Chute de ponte
- [ ] Retard de croissance
- [ ] Autres

Précisez

Choix du traitement AB de 1 ère intention

- [ ] historique de l'élevage (profil de résistance aux AB dans les bandes précédentes)
- [ ] après antibiogramme direct (rapide-1 jour)
- [ ] selon l'expérience
- [ ] selon l'âge des sujets
- [ ] selon le prix
- [ ] selon la disponibilité
- [ ] autres

Précisez

Mode d'administration
avec 1 le plus utilisé

1st choice

- ○ PO
- ○ IM
- ○ Nébullisation

2nd choice

- ◯ PO
- ◯ IM
- ◯ Nébullisation

3rd choice

- ◯ PO
- ◯ IM
- ◯ Nébullisation

Durée générale des prescriptions

selectionné un ou plusieurs

- ☐ 1 jour
- ☐ 2 jours
- ☐ 3 jours
- ☐ 4 jours
- ☐ 5 jours
- ☐ 6 jours
- ☐ 7 jours
- ☐ > 7 jours

Utilisation des antibiotiques en chimioprévention

sur des sujets indemnes pour prévenir les maladies

- ◯ Rarement
- ◯ Couramment
- ◯ Jamais

Prescription d'AB en hors AMM

- ◯ Oui
- ◯ Non

Raisons de préscription en hors AMM

selectionner une ou plusieurs

- ☐ Absence d'alternatives thérapeutiques
- ☐ Efficacité
- ☐ Prix
- ☐ Adaptation à la résistance bactérienne
- ☐ Autres

Précisez

____

Laboratoires, antibiorésistance et gestion des déchets biologiques

Recours aux laboratoires de bactériologie

- ◯ Rarement
- ◯ Fréquement
- ◯ Jamais

Pourquoi

- ☐ Distance
- ☐ Temps résultat-Urgence des cas
- ☐ Frais
- ☐ Absence de labo à proximité
- ☐ Refus de l'éleveur
- ☐ Autres

Disposez-vous d'un labo (réalisation des antibiogrammes dans votre cabinet)

- ◯ Oui
- ◯ Non

Gestion des Déchets du labo

Boite de péteri+ consommables...

- ☐ poubelle spéciale
- ☐ déchets ménagères
- ☐ Traitement (désinfectant)+déchets ménagères
- ☐ Traitement + poubelle spéciale
- ☐ Autres

Précisez

Isolement de bactéries multirésistantes

- ◯ Rarement
- ◯ Fréquement
- ◯ Jamais

Citez les plus rencontrées

Gestion des flacons vides et/ou périmés

- ☐ poubelle spéciale
- ☐ déchets ménagères
- ☐ autres

précisez

Gestion des cadavres

- *Blouze jetable et gants*

- [ ] incinération
- [ ] enfouis dans l'élevage
- [ ] déchets ménagères
- [ ] laissés à l'éleveur
- [ ] autres

Précisez

» Les 3 bactéries les plus isolées + les Antibiotiques aux quels elles sont résistantes

Bactérie N°1:Nom & Antibiotiques aux quels elle est résistante

---

Bactérie N°2: Nom & Antibiotiques aux quels elle est résistante

---

Bactérie N°3: Nom & Antibiotiques aux quels elle est résistante

---

Automédication

Automédication dans votre clientele

- ( ) rarement
- ( ) fréquement
- ( ) jamais

Causes de l'automédication

- [ ] Facilité d'accés aux antibiotiques
- [ ] Pression économique
- [ ] Méconnaissance des risques
- [ ] autres

précisez

---

causes de disponibilité des AB

- [ ] Vente sans ordonnance
- [ ] Contrebande
- [ ] autres

précisez
