# Supplementary material for: Antibiotic use in poultry farming: a cross-sectional study of veterinary practices in Tunisia
Source: Front Antibiot. 2025 Oct 14;4:1646766. doi: 10.3389/frabi.2025.1646766 (PMC12558877; doi:10.3389/frabi.2025.1646766)
Supplement: Supplementary file 2 [file DataSheet2.pdf]

Use of Antibiotics in Avian Veterinary Medicine

General Information

Gender

- Man
- Woman

Place of Work

- Private Practice
- Breeding Company (or Holding)
- OTD (Technical Poultry Organization)
- Other
  Please specify: ________

Year of Diploma/Graduation

Number of Years of Experience in Avian Medicine

Training on Antibiotic Resistance

- Yes
- No

Number of training days: ________

Year of the most recent training Training Organization

List of the 3 Most Used Antibiotics

Antibiotic 1: Name & Approximate Quantity Prescribed Per Year
Please specify the unit

===== Page 2 =====

Antibiotic 2: Name & Approximate Quantity Prescribed Per Year
Please specify the unit

Antibiotic 3: Name & Approximate Quantity Prescribed Per Year
Please specify the unit

Factors Influencing the Treatment Plan

Symptoms for which the use of antibiotics is systematic
Select one or more

- Respiratory
- Digestive
- Nervous
- Drop in Egg Production

- Growth Retardation
- Others
  Please specify: ________

Choice of First-Line Antibiotic Treatment

- History of the farm (antibiotic resistance profile in previous flocks)
- After direct antibiogram (rapid - 1 day)
- Based on experience
- According to the age of the subjects
- According to price
- According to availability
- Others
  Please specify: ________

Method of Administration
With 1 being the most used

1st choice

- PO (Oral)
- IM (Intramuscular)
- Nebulization

===== Page 3 =====

2nd choice

- ○ PO (Oral)
- ○ IM (Intramuscular)
- ○ Nebulization

3rd choice

- ○ PO (Oral)
- ○ IM (Intramuscular)
- ○ Nebulization

General Duration of Prescriptions
Select one or more

- 1 day

- 2 days
- 3 days
- 4 days
- 5 days
- 6 days
- 7 days
- > 7 days

Use of antibiotics for chemoprevention
On healthy subjects to prevent diseases

- o Rarely
- o Commonly
- o Never

Prescription of Antibiotics Off-Label

- o Yes
- o No

Reasons for Off-Label Prescription
Select one or more

- Lack of therapeutic alternatives
- Effectiveness
- Price
- Adaptation to bacterial resistance
- Others
  Please specify: _________

===== Page 4 =====

Laboratories, Antibiotic Resistance, and Biological Waste Management

Use of Bacteriology Laboratories

- Rarely
- Frequently
- Never

Why

- Distance
- Result Time - Urgency of Cases
- Cost
- Lack of a nearby laboratory
- Refusal by the farmer
- Others
  Please specify: ________

Do you have a laboratory (performing antibiograms in your practice)?

- Yes
- No

Laboratory Waste Management
Petri dishes, consumables...

- Special bin
- Household waste
- Treatment (disinfectant) + household waste
- Treatment + special bin
- Others
  Please specify: ________

Isolation of Multi-resistant Bacteria

- Rarely
- Frequently
- Never

List the most commonly encountered ones: ________

Management of Empty and/or Expired Vials/Bottles

- Special bin
- Household waste
- Others
  Please specify: ________

===== Page 5 =====

Please specify: ________

Carcass Management

Disposable overalls and gloves

- Incineration
- Buried on the farm
- Household waste
- Left to the farmer
- Others
  Please specify: _________

» The 3 Most Isolated Bacteria + The Antibiotics to which they are Resistant

Bacterium No. 1: Name & Antibiotics to which it is resistant

Bacterium No. 2: Name & Antibiotics to which it is resistant

Bacterium No. 3: Name & Antibiotics to which it is resistant

Self-Medication

Self-medication among your clientele

- Rarely
- Frequently
- Never

Causes of self-medication

- Ease of access to antibiotics
- Economic pressure
- Lack of awareness of the risks
- Others
  Please specify: _________

===== Page 6 =====

Causes of antibiotic availability [for self-medication]

- Sale without prescription
- Smuggling
- Others
  Please specify: _________

[File Content End]
